# Inactivation of Carbonyl-Detoxifying Enzymes by H_2_O_2_ Is a Trigger to Increase Carbonyl Load for Initiating Programmed Cell Death in Plants

**DOI:** 10.3390/antiox9020141

**Published:** 2020-02-06

**Authors:** Md. Sanaullah Biswas, Ryota Terada, Jun’ichi Mano

**Affiliations:** 1 Department of Horticulture, Bangabandhu Sheikh Mujibur Rahman Agricultural University, Gazipur 1706, Bangladesh; 2 Faculty of Agriculture, Yamaguchi University, Yoshida 1677-1, Yamaguchi 753-8515, Japan; y.rt.univ028.ge78@gmail.com; 3 Science Research Center, Organization of Research Initiatives, Yamaguchi University, Yamaguchi 753-8511, Japan; 4 Graduate School of Science and Technology for Innovation, Yamaguchi University, Yamaguchi 753-8511, Japan

**Keywords:** lipid peroxide, oxidative stress, oxylipin, reactive carbonyl species (RCS), reactive electrophile species (RES), redox signal, ROS

## Abstract

H_2_O_2_-induced programmed cell death (PCD) of tobacco Bright Yellow-2 (BY-2) cells is mediated by reactive carbonyl species (RCS), degradation products of lipid peroxides, which activate caspase-3-like protease (C3LP). Here, we investigated the mechanism of RCS accumulation in the H_2_O_2_-induced PCD of BY-2 cells. The following biochemical changes were observed in 10-min response to a lethal dose (1.0 mM) of H_2_O_2_, but they did not occur in a sublethal dose (0.5 mM) of H_2_O_2_. (1) The C3LP activity was increased twofold. (2) The intracellular levels of RCS, i.e., 4-hydroxy-(*E*)-hexenal and 4-hydroxy-(*E*)-nonenal (HNE), were increased 1.2–1.5-fold. (3) The activity of a reduced nicotinamide adenine dinucleotide phosphate (NADPH)-dependent carbonyl reductase, scavenging HNE, and *n*-hexanal was decreased. Specifically, these are the earliest events leading to PCD. The proteasome inhibitor MG132 suppressed the H_2_O_2_-induced PCD, indicating that the C3LP activity of the *β*1 subunit of the 20S proteasome was responsible for PCD. The addition of H_2_O_2_ to cell-free protein extract inactivated the carbonyl reductase. Taken together, these results suggest a PCD-triggering mechanism in which H_2_O_2_ first inactivates a carbonyl reductase(s), allowing RCS levels to rise, and eventually leads to the activation of the C3LP activity of 20S proteasome. The carbonyl reductase thus acts as an ROS sensor for triggering PCD.

## 1. Introduction

Reactive oxygen species (ROS), such as superoxide radical and hydrogen peroxide (H_2_O_2_), are metabolic outcomes of various cellular processes in plants. Versatile biological actions of ROS, collectively termed as oxidative signals, largely depends on the ROS concentration [1]. Recent evidence shows that ROS are involved in diverse signaling functions in plants, from cell cycle regulation to whole plant senescence [2]. The transition from cellular proliferation to cell elongation, which is necessary for plant growth in the early stages of cell differentiation, is regulated by ROS signals [3]. The levels of superoxide radical and H_2_O_2_ are high in the elongation and meristematic zone of Arabidopsis root [4]. ROS also play an important role in seed germination; superoxide and H_2_O_2_ are increased in the radicle of Arabidopsis during germination [5]. Thus, ROS signals are necessary in various stages of plant growth and development.

Programmed cell death (PCD) is an essential phenomenon in plants for maintaining their growth and development. In many cases, PCD is started by the rise of ROS levels, which is stimulated by various endogenous or exogenous stimuli. Aleurone layer of cereal grains and the lateral root cap cells are eliminated as a result of PCD with a rise of H_2_O_2_ level [6,7]. Under water deficit condition rice developing anthers started PCD due to enhancement of H_2_O_2_ level and down-regulation of antioxidant transcripts [8]. In response to salinity and sorbitol stress, tobacco BY-2 cells increase the level of superoxide radical, which triggers PCD [9]. High temperature causes a rise in the H_2_O_2_ level in tobacco BY-2 cells before initiation of PCD [10]. H_2_O_2_ is also involved in the hypersensitive response (HR)-like cell death in tobacco leaves [11]. However, the mechanism of ROS action to initiate PCD was largely unclear. 

Recently, we found that, in the mechanism by which ROS initiates PCD, the lipid peroxide-derived reactive carbonyl species (RCS) play critical roles in conveying ROS signals [12]. RCS is a group name of the α,β-unsaturated aldehydes and ketones, such as acrolein and 4-hydroxy-(*E*)-2-nonenal (HNE) [13,14]. RCS are generated from lipid peroxides (LOOH) and act as agents to mediate ROS signal to target proteins in the heat shock-responsive gene regulation [15], senescence [16], abscisic acid (ABA) signaling for stomatal closure [17,18], and auxin signaling for lateral root formation [19] in plants. We reported that tobacco BY-2 cells exposed to H_2_O_2_ generated acrolein and HNE, and a chemical carbonyl scavenger prevented the initiation of PCD, without affecting the increase in the intracellular ROS level [12]. 

We further revealed that the targets of RCS were two caspase-like proteases [20]. Caspases are cysteine-containing aspartate-specific proteases that are involved in the initiation of PCD in animals. Plants do not have proteins homologous to animal caspases but have caspase-like proteases that show the same substrate specificity for the artificial peptides to caspase-1 and caspase-3. Vacuolar processing enzyme (VPE), an ortholog of asparaginyl endopeptidase (legumain), shows the caspase-1-like protease (C1LP) activity and is responsible for processing vacuolar proteins [21]. The functions of C1LP in plants and caspase-1 in animals to determine cell fate are comparable [22]. Activation of C1LP is associated with the aluminum-induced PCD in the root tip of a peanut [23]. The caspase-3-like protease (C3LP) is also involved in the initiation of PCD in cadmium-induced PCD [24] and hypersensitive response-induced PCD [25] in *Arabidopsis thaliana*. High temperature treatment to tobacco BY-2 cells increases the H_2_O_2_ level, followed by the activation of C3LP before PCD occurs [10]. In our previous study [20], we found that H_2_O_2_ addition to BY-2 cells activated both C3LP and C1LP before PCD symptoms become apparent. The addition of acrolein and HNE to the cell-free extract rapidly activated both C3LP and C1LP, but the addition of H_2_O_2_ to the extract failed to activate the proteases. These results indicated that, in BY-2 cells, the RCS accumulating due to the H_2_O_2_ stimulus activated C3LP and C1LP and started PCD. Thus, the activation of C3LP and C1LP was ascribed to RCS that was generated by the oxidative stimulus. 

We tentatively concluded that C3LP was more relevant than C1LP to the initiation of PCD because, on addition of lethal dose of acrolein, the activation of the former was more rapid and larger than that of the latter [20]. However, the contribution of C1LP to the H_2_O_2_-induced PCD remains open. It is also unclear which protein is responsible for the RCS-stimulated activity of C3LP because two distinct proteins have been known to show C3LP activity: the *β*1 subunit of the 20S proteasome (PBA1) [25,26] and cathepsin B [27].

Another critical question is about the type of RCS that activated C3LP. In our previous study, we analyzed carbonyls in the cells 2 h after H_2_O_2_ addition and found that at least eight types of oxylipin carbonyls were increased [12]. The two potent RCS acrolein and HNE, found as the increasing carbonyls, showed the ability to activate C3LP and C1LP in vitro [20]. On the other hand, C3LP was activated within 30 min after H_2_O_2_ addition. It is therefore expected that certain types of RCS should be increased prior to the C3LP activation on the addition of H_2_O_2_ to BY-2 cells. 

In this study, attempting to elucidate the detailed mechanism of the H_2_O_2_-induced PCD of BY-2 cells, we monitored early biochemical events in response to oxidative stimulus. We confirmed that C3LP is critical for the PCD, and found its activity is attributable to PBA1. In response to H_2_O_2_, BY-2 cells accumulated HNE and 4-hydroxy-(*E*)-hexenal (HHE) in 10 min. Interestingly, at the same time, an NADPH-dependent carbonyl reductase(s), showing HNE-reducing activity, was largely inactivated. The same activity in cell-free extract was inactivated by the addition of H_2_O_2_, indicating that H_2_O_2_ attenuated the carbonyl reductase activity to enhance the RCS level. We propose a new mechanism of ROS-induced PCD, where the carbonyl reductase(s) acts as an ROS sensor for initiating the PCD process.

## 2. Materials and Methods 

### 2.1. Culture of Cells

Cell suspension of tobacco BY-2 (*Nicotiana tabacum* L. cv. Bright Yellow-2) (RIKEN BioResource Research Center, Tsukuba, Japan) were cultured in Murashige and Skoog medium (MP Bio, Tokyo, Japan) supplemented with sucrose (30 g L^−1^), myo-inositol (100 mg L^−1^), KH_2_PO_4_ (200 mg L^−1^), thiamine HCl (0.5 mg L^−1^), and 2,4-dichlorophenoxyacetic acid (0.2 mg L^−1^) (all these chemicals were purchased from FUJIFILM Wako Pure Chemical, Osaka, Japan) at pH 5.6. Cells were propagated with continuous rotation at 120 rpm in darkness at 25 °C. Every seven days, 0.5 mL dense cells were sub-cultured to a fresh medium (50 mL). The exponential growth phase cells at the fourth day were selected for experiment [28]. H_2_O_2_ was added to the culture medium (H_2_O_2_ treatment). After an incubation, the cells were harvested and washed with distilled water for analyses. 

### 2.2. Cell Viability Assay

Cell viability was determined by fluorescein diacetate (FDA) staining. Harvested cells were incubated in FDA solution (1 µg mL^–1^) for 5 min, washed twice with distilled water, and then observed under a fluorescence microscope (Leica AF6000, Wetzlar, Germany). Viable cells cleave FDA to form fluorescein, which fluoresce with excitation at 485 nm and emission at 515 nm. Dead cells do not fluoresce. 

### 2.3. Determination of C1LP and C3LP Activities 

Protein extract from BY-2 cells was prepared as described previously [20,21]. The harvested cells were frozen with liquid nitrogen and ground to powder with a mortar and pestle. Then, the ground powder of cells was transferred to a centrifuge tube and immediately added the protease extraction medium containing 50 mM sodium acetate, pH 5.5, 50 mM NaCl, 1 mM EDTA, 1 mM phenylmethylsulfonyl fluoride (PMSF), and 0.1 mM E64-d (a thiol protease inhibitor). After centrifugation at 14,000× *g* for 30 min at 4 °C, supernatant was collected as the protein extract.

The tetrapeptide fluorogenic substrates Ac-YVAD-AMC for C1LP and Ac-DEVD-AMC for C3LP and the respective inhibitors (Ac-YVAD-CHO and Ac-DEVD-CHO) were purchased from Peptide Institute (Osaka, Japan). For measuring a protease activity, two tubes were prepared. One had a reaction mixture (0.2 mL) containing the protease sample (50 µL of protein extract or 50 µg of protein) in 20 mM Na-acetate, pH 5.5, 0.1 M dithiothreitol, 0.1 mM EDTA, and 1 mM PMSF with an inhibitor at 0.1 mM, and the other had the same reaction mixture without the inhibitor. They were preincubated at 37 °C for 1 h. Then, the substrate peptide was added to each tube at 0.1 mM. After an incubation at 37 °C for 1 h, the AMC released from the substrate was determined with a fluorescence microplate reader (Twincle LB970, Berthold Japan, Tokyo, Japan) (excitation 380 nm; emission 445 nm). The specific activity of proteases was calculated from the difference between the absence and the presence of an inhibitor. A standard curve was developed using a series of AMC (Peptide Institute) solutions in the 0 nM to 200 nM range. 

In vitro activation of C1LP and C3LP activity was done by the addition of either acrolein or H_2_O_2_ to protein extract. After incubation, the protein extract was allowed passage through a PD MiniTrap G-25 column (GE Healthcare, Tokyo, Japan), equilibrated with protease extraction medium, to remove small molecules. A 0.2 mL of reaction mixture with the eluted protein extract was prepared similarly as described above. 

### 2.4. Analysis of Glutathione

Total glutathione (GSH + oxidized form (GSSG)) was determined as described previously [20,29] with a minor modification. Briefly, harvested cells (about 0.4 g) were immediately ground in liquid nitrogen with mortar and pestle and suspended in two volumes of cold 5% sulphosalicylic acid in a 1.5 mL centrifuge tube. For glutathione analysis, the supernatant was collected after centrifugation at 20,000× *g* for 15 min at 4 °C. The cell extract (a 0.1 mL aliquot) was neutralized by mixing with 0.9 mL of 0.1 M HEPES-KOH, pH 7.4, containing 5 mM EDTA. The mixture was divided into two fractions for determining total GSH (GSH + oxidized form (GSSG)) and GSSG. A 1.0 mL reaction mixture was prepared with 0.3 mL of neutralized cell extract containing 0.1 M HEPES-KOH, pH 7.4, 5 mM EDTA, 10 mM 5,5’-dithiobis-2-nitrobenzoic acid (DTNB), 0.5 unit glutathione reductase. The reaction was started with the addition of 0.2 mM NADPH, and the rate of absorbance increase at 412 nm was recorded for 1 min. The GSSG content was determined after masking GSH by 20 μL of 2-vinylpyridine to the neutralized cell extract. The emulsified residual 2-vinylpyridine was removed through a brief centrifuge; then, GSSG was determined, as described above, for total GSH. The GSH content was estimated after the subtraction of GSSG to the total GSH. A GSH standard curve was prepared with authentic GSH (Wako, Japan) in the range of 0–0.1 mM. 

### 2.5. Analysis of Ascorbate

Asc and dehydroascorbic acid (DHA) were determined as described previously [20,30] with some modifications. The cells were extracted with the same protocol of GSH analysis. The total ascorbate (Asc + DHA) content was estimated in a 1 mL reaction mixture containing 0.1 mL aliquot of cell extract, 0.2 mL of 150 mM phosphate, pH 7.4, containing 5 mM EDTA, and 0.05 mL of 10 mM dithiothreitol (DTT). To remove excess DTT, 0.05 mL of 0.5% *N*-ethylmaleimide was added after 10-min incubation at room temperature. To determine Asc, 0.1 mL of water was added in the reaction mixture instead of DTT and *N*-ethylmaleimide. Then, the reaction mixture was incubated at 40 °C for 40 min with 0.2 mL of 10% trichloroacetic acid, 0.2 mL of 44% ortho-phosphoric acid, 0.2 mL of 4% α,α-dipyridyl in 70% ethanol, and 0.3% (*w*/*v*) FeCl_3_ for color development. The absorbance at 525 nm was measured, and the concentration of DHA was calculated from the difference of total ascorbate (Asc + DHA) and Asc. A standard curve was prepared with Asc in the 0–250 µM range.

### 2.6. Detection and Quantification of Carbonyls Using HPLC

The fresh cells (0.4 g) were filtered and washed twice with distilled water, then immediately transferred into a screw-capped glass tube containing 2.5 mL of acetonitrile containing internal standard (25 nmol of 2-ethylhexanal) and 0.005% (*w*/*v*) butylhydroxytoluene to prevent autoxidation of unsaturated organic compounds. Then, the tube was closed and incubated in a water bath at 60 °C for 30 min. Avoiding cell debris, only clear solution was collected by Pasteur pipette to another glass tube, and the carbonyls were derivatized with 2,4-dinitrophenylhydrazine (0.5 mM) and formic acid (0.5 M) for 60 min at 25 °C. Then, the formic acid in the solution neutralized with 0.45 g of NaHCO_3_ in 2.5 mL of saturated NaCl. After thorough mixing and subsequent centrifugation, the upper layer was collected, dried, and dissolved in a smaller volume of acetonitrile. The hydrazone-derivatized carbonyls were determined on a reverse phase (C30) HPLC [12,17,31,32].

### 2.7. Determination of Enzymatic Activity

Proteins were extracted as follows. The cells harvested and washed on a nylon mesh were frozen with liquid nitrogen and ground to powder with a mortar and pestle. About 0.4 g powdered cells were transferred to the reductase extraction medium containing 50 mM KH_2_PO_4_, pH 7.4, 1 mM EDTA, and 1×Protease Inhibitor Cocktail (Sigma-Aldrich Japan, Tokyo, Japan) and extensively mixed. After centrifugation with 10,000× *g* for 10 min at 4 °C, 0.5 mL cell extract was allowed passage of the PD MiniTrap G-25 column equilibrated with reductase extraction medium. Protein content was determined with the Protein Assay CBB solution (Nacalai Tesque, Kyoto, Japan) with bovine serum albumin as the standard. 

The aldehyde reducing activity by the reductases of the protein extract was measured using 50 mM Mes-NaOH, pH 6.0, containing 0.1 mM NADPH. Various species of aldehydes (HNE, *n*-hexanal, acetaldehyde, butyraldehyde, (*Z*)-3-Hexenal) were used as the substrate of reductases and the rate of oxidation of NADPH was measured at 340 nm using a Shimadzu (Kyoto, Japan) MPS-2000 spectrophotometer.

## 3. Results

### 3.1. Threshold Level of Hydrogen Peroxide for Inducing PCD

**Figure 1:**
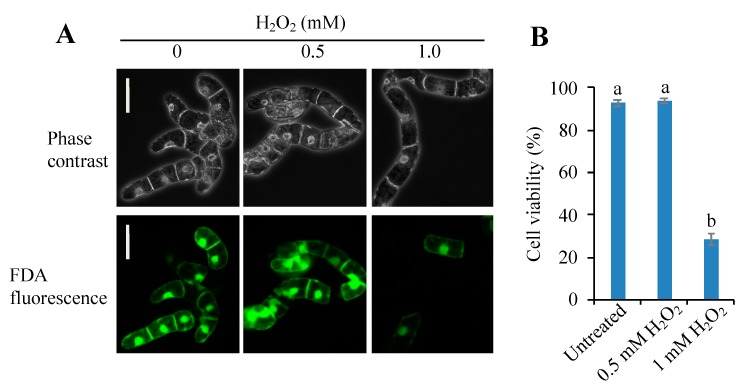
Effects of H_2_O_2_ concentration on the viability of Bright Yellow-2 (BY-2) cells. A 0.5 mL aliquot of cells from 7-d culture were sub-cultured in 50 mL of fresh culture medium and, after 4 d, the culture medium was supplemented with H_2_O_2_ to 0.5 mM or 1 mM. After 5 h incubation cells were harvested and stained with fluorescein diacetate (FDA). Living cells and dead cells were counted as described in the Materials and Methods section. Cells forming a single layer under microscopy were chosen for evaluation. (**A**) Typical phase-contrast (top) and FDA-fluorescence (bottom) images of the same field. Bar = 50 µm. (**B**) Percentage of cell viability. The total number of cells was counted under phase contrast observation, and the FDA-stained cells were counted under fluorescence observation. A total of 200 cells were observed in each treatment. Means ± SE of three independent experiments are shown. Differences among treatments were analyzed by Tukey’s test: *p* < 0.05. Different letters among the treatments represent statistically significant differences.

Because addition of H_2_O_2_ to BY-2 cells caused a rapid increase (within 10 min) in C3LP and C1LP [20], it is expected that certain types RCS are generated in the cells. On the other hand, addition of H_2_O_2_ is generally oxidative stimulus, and it will cause lipid peroxidation, leading to the formation of various RCS. We attempted to distinguish the RCS that are responsible for the triggering of PCD from those generated as a consequence of the progress of oxidative stress. For this purpose, we have set up two H_2_O_2_ treatment conditions, i.e., lethal and sublethal. BY-2 cells were treated with H_2_O_2_ at various concentrations, and it was found that 1 mM H_2_O_2_ induced about 75% cell death in 5-h incubation, while 0.5 mM H_2_O_2_ did not increase cell death as compared with untreated control (Figure 1). Specifically, 1 mM H_2_O_2_ was a lethal dose to induce cell death and 0.5 mM was sublethal. The biochemical events that triggered PCD thus should have occurred earlier than 5 h only in the cells treated with 1 mM H_2_O_2_ but should not in those treated with 0.5 mM H_2_O_2_.

### 3.2. Caspase-Like Proteases in Tobacco BY-2 Cells Are Activated Only When H_2_O_2_ Induces PCD

**Figure 2:**
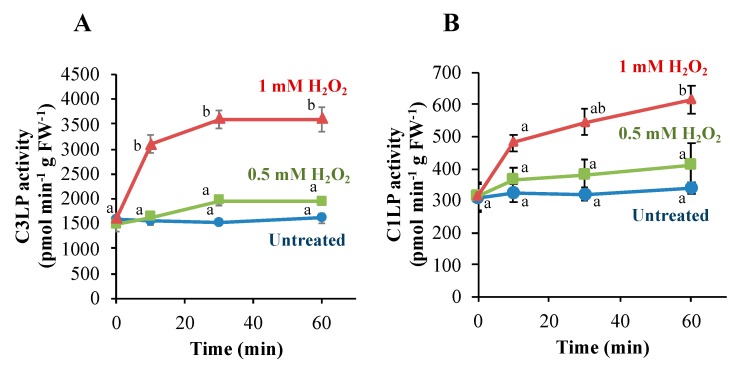
Activation of caspase-3-like protease (C3LP) (**A**) and caspase-1-like protease (C1LP) (**B**) in BY-2 cells after exposure to H_2_O_2_. Four-d cultured cells were treated with H_2_O_2_ to 0.5 mM or 1 mM, as in Figure 1. Proteins was extracted from the cells as described in the Material and Methods section. The activity of C3LP (**A**) and C1LP (**B**) was measured with the respective substrates Ac-DEVD-AMC and Ac-YVAD-AMC, as in the Materials and Methods section. The data are the mean ± SE. Different letters represent significantly different values (*p* < 0.05 on Tukey test).

The addition of H_2_O_2_ to BY-2 cells at a lethal dose activated C3LP and C1LP within 30 min [20]. To compare the effects of sublethal and lethal doses of H_2_O_2_, we treated BY-2 cells with H_2_O_2_ at 0.5 and 1 mM and determined the caspase activities (Figure 2). Untreated cells exhibited constitutive levels of C3LP and C1LP activity. H_2_O_2_ at the lethal dose (1 mM) increased C3LP significantly in 10 min, and after 30 min, the activity reached 2.5-fold. A sublethal dose of H_2_O_2_ (0.5 mM) did not increase the C3LP activity (Figure 2A). By the lethal dose of H_2_O_2_, the C1LP activity was also increased to a significantly higher level in 60 min, while in 10 min, the increase was apparently smaller (Figure 2B). The sublethal dose of H_2_O_2_ caused a very slight increase in the C1LP activity. Thus, early activation of C3LP and C1LP was consistently associated with the later occurrence of PCD.

### 3.3. Activation of C3LP, Rather Than C1LP, Triggers PCD

**Figure 3:**
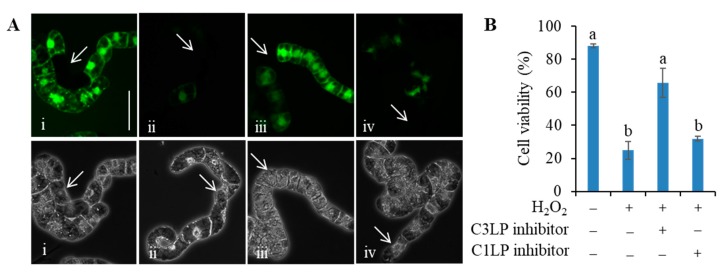
Effects of caspase inhibitors on the H_2_O_2_-induced cell death. Four-d cultured BY-2 cells were treated, as in Figure 1, with 1 mM H_2_O_2_ in the presence or absence of an inhibitor. The cells were harvested after 5-h incubation, and FDA-stained cells were counted as living cells. (**A**) Typical fluorescence images (top) and phase contrast images (bottom) of the same field: (i) untreated cells as control, (ii) 1 mM H_2_O_2_, (iii) 1 mM H_2_O_2_ + 20 µM C3LP inhibitor, and (iv) 1 mM H_2_O_2_ + 20 µM C1LP inhibitor (top). White arrows indicate dead cells. Bar = 50 µm. (**B**) Percentage of living cells. A total of 400 cells were observed in each treatment. Mean of 3 runs ± SEM. Different letters represent significantly different values (*p* < 0.05 on Tukey test).

The activation of C3LP in response to a lethal dose of H_2_O_2_ was more rapid and to a larger extent than C1LP (Figure 2). To evaluate the contribution of each proteases to PCD initiation, we treated the cells with the inhibitors specific to C3LP or C1LP and examined whether one of them or both prevents H_2_O_2_-induced PCD or not (Figure 3). The H_2_O_2_ treatment caused PCD in 75% cells in 5 h. The addition of C3LP inhibitor efficiently suppressed H_2_O_2_-induced cell death; only 25% cells were dead. On the other side, C1LP inhibitor could not prevent H_2_O_2_-induced cell death effectively; 70% cells were dead. We also tested whether these caspase inhibitors prevent the PCD induced by 0.2 mM acrolein. The addition of acrolein at 0.2 mM induced 90% cell death after 5-h incubation (Figure S1). Addition of the C3LP inhibitor prior to the acrolein treatment suppressed the cell death to 30%, as compared with 80% death for the acrolein-treatment without protease inhibitors. On the other side, the C1LP inhibitor did not efficiently prevent acrolein-induced cell death. These results suggest that the activation of C3LP is critical for the PCD initiated by oxidative stimuli.

### 3.4. The C3LP Activity of the 20S Proteasome Is Responsible for the H_2_O_2_-Initiated PCD 

**Figure 4:**
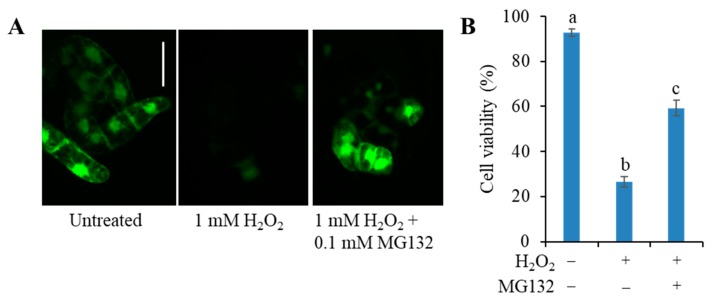
Effect of MG132 on the cell survival in H_2_O_2_-treated tobacco BY-2 cells. Four-d cultured cells were treated with either 1 mM H_2_O_2_ or 1 mM H_2_O_2_ plus 0.1 mM MG132. MG132 was added 30 min before addition of H_2_O_2_. After 5-h incubation, cell death was determined with FDA staining, as in Figure 1. (**A**) Typical photographs are shown: untreated cells as control (left), treated with H_2_O_2_ at 1 mM (middle), and H_2_O_2_ at 0.2 mM plus 0.1 mM MG132 (right). Bar = 50 µm. (**B**) Cell viability (%). All values are mean ± SE, and the data represent three independent experiments. Different letters represent significantly different values (*p* < 0.05 on Tukey test).

Two distinct proteins are known to show the C3LP activity, i.e., PBA1, a subunit of the 20S proteasome [25,26,33] and cathepsin B [27]. Vacca et al. [34] measured ROS accumulation and C3LP in the heat shocked tobacco BY-2 cells in absence and presence of the proteasome inhibitor MG132. They found that MG132 prevented PCD and specifically decreased ROS level and C3LP activity. To clarify which protein is really involved in the H_2_O_2_-induced PCD, we tested the effect of MG132. BY-2 cells were pre-incubated with MG132 at 0.1 mM for 30 min, and then H_2_O_2_ was added at 1 mM to induce PCD. H_2_O_2_ at 1 mM in 5-h incubation induced about 75% cell death, and proteasome inhibitor MG132 significantly reduced cell death to 40% (Figure 4). Thus, the C3LP activity responsible for PCD was attributed to the β1 subunit of the 20S proteasome. 

### 3.5. Limited Types of Oxylipin Carbonyls Are Increased in Very Early Stages of PCD

We presume that oxylipin carbonyls generated in the H_2_O_2_-stimulated cells activate C3LP and C1LP and thereby initiate PCD [20]. Because C3LP is activated rapidly on H_2_O_2_ addition (Figure 2), the oxylipin carbonyls to activate it should also be rapidly increased. 

**Figure 5:**
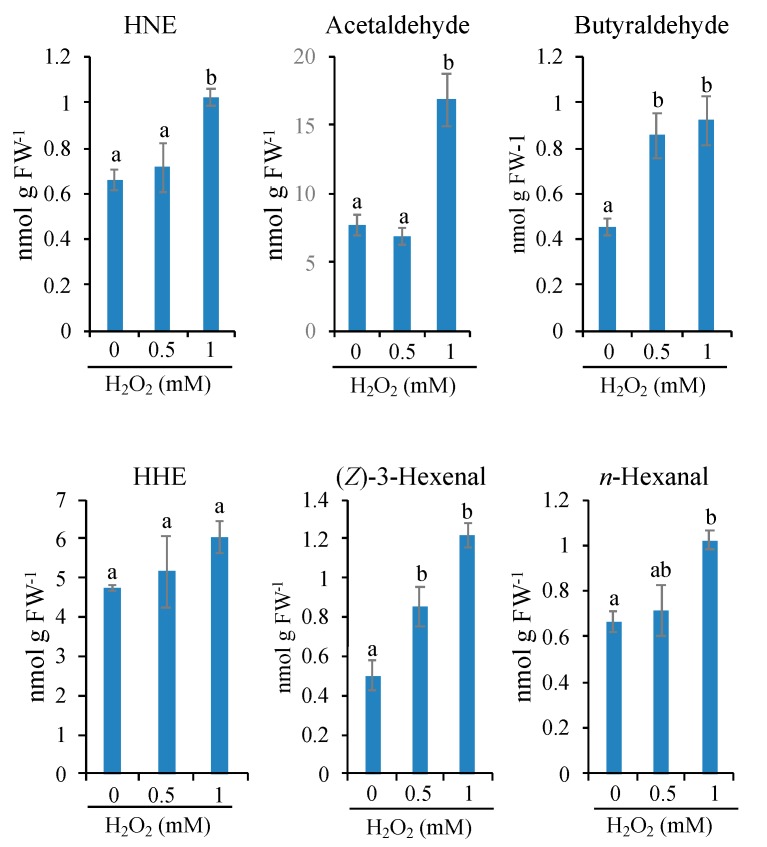
Effects of H_2_O_2_ on the carbonyl levels in BY-2 cells. To 4-d-cultured cells, H_2_O_2_ was added to the indicated concentration. After a 10-min incubation, cells were harvested, and the intracellular contents of carbonyl levels were determined as in the Materials and Methods section. Results of 4-hydroxy-(*E*)-2-nonenal (HNE), acetaldehyde, butyraldehyde, 4-hydroxy-(*E*)-hexenal (HHE), 3-(Z)-hexanal and *n*-hexanal are shown. Each point represents the mean of three independent experiments and the error bars the SEM. Different letters represent significantly different values (*p* < 0.05 on Tukey test).

BY-2 cells were treated with the lethal (1 mM) and sublethal (0.5 mM) dose of H_2_O_2_, and after 10-min incubation, oxylipin carbonyls were extracted and quantified on HPLC (Figure S2). We found that the lethal dose of H_2_O_2_ increased several types of RCS and other carbonyls as compared with their basal levels in the untreated samples (Figure 5). Among the RCS, HNE increased significantly. HHE also showed an increasing trend although the difference was insignificant. The non-RCS carbonyls acetaldehyde, butyraldehyde, (*Z*)-3-hexenal, and *n*-hexanal were also increased significantly (Figure 5). In the cells treated with the sublethal dose of H_2_O_2_ (0.5 mM), HNE, HHE, acetaldehyde, and *n*-hexanal were not changed, but butyraldehyde and (*Z*)-3-hexenal were increased twofold compared with the untreated cells (Figure 5). When the lethal and sublethal conditions are compared, only limited types of oxylipin carbonyls, i.e., HNE (a RCS), acetaldehyde, and *n*-hexanal (non-RCS carbonyls), were increased. It is expected that these carbonyls are relevant to the C3LP activation and initiation of PCD. 

HNE and *n*-hexanal have been known to induce PCD in tobacco BY-2 cells [12], and HNE can directly activate C3LP [20]. Here, we tested the ability of acetaldehyde for the activation of C3LP (Figure S3). Four-d cultured cells were treated with various concentrations of acetaldehyde, from 0.5 mM to 10 mM, and then measured for C3LP activity. Acetaldehyde up to 3 mM did not increase C3LP, but at 10 mM, it significantly increased the C3LP activity compared with that in the untreated cells. Thus, the three oxylipin carbonyls that were increased rapidly on the addition of lethal dose of H_2_O_2_ were capable of activating C3LP.

### 3.6. Effects of H_2_O_2_ on the Intracellular Glutathione and Ascorbate Levels 

**Figure 6:**
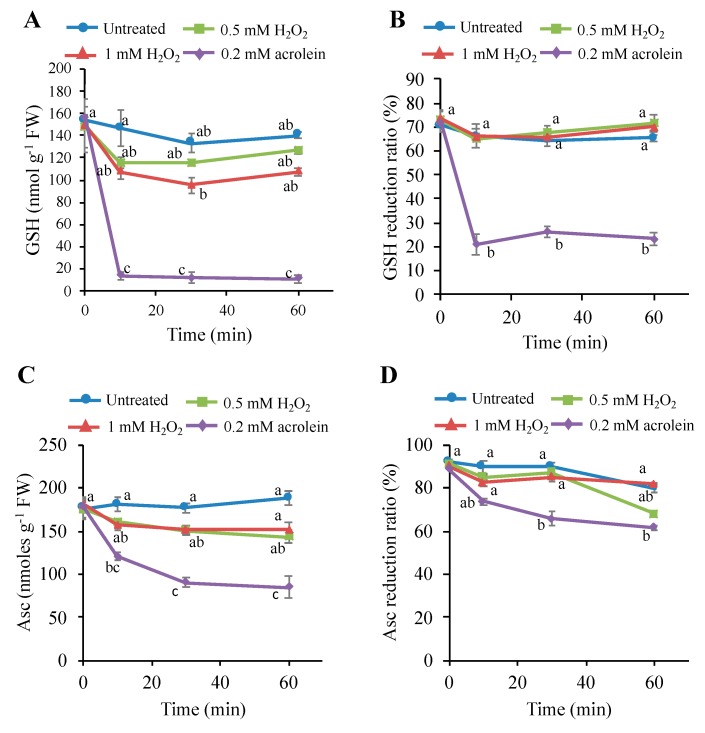
Changes in the contents and reduction ratios of ascorbate (Asc) and glutathione (GSH) in tobacco BY-2 cells treated with H_2_O_2_ or acrolein. H_2_O_2_ and acrolein at the indicated concentration was added to 4-d cultured cells, and then Asc and GSH contents were measured as described in the Materials and Methods section. (**A**) Reduced Asc, (**B**) Asc reduction ratio (%), (**C**) contents of reduced GSH, and (**D**) GSH reduction ratio (%). Each point represents the mean of three independent experiments and the error bars of the SEM. Different letters represent significant different data (*p* < 0.05 on Tukey test).

A possible cause of the RCS increases is the depletion of glutathione (GSH) in the cells because the thiol moiety of GSH provides a major defense against the various electrophiles, including RCS [35,36]. Consumption of ascorbate by H_2_O_2_ also may facilitate the loss of GSH pool and can be an indirect cause of RCS increase. To measure early changes in the GSH and ascorbate status, tobacco BY-2 cells were treated with a lethal and sublethal doses of H_2_O_2_ and sampled at different time points (Figure 6). Lethal level of H_2_O_2_ (1 mM) decreased total glutathione pool (GSH + GSSG) by 40% in 30 min. In 60-min incubation, this lowered level of GSH recovered by about 10% (Figure 6A). The GSH reduction ratio was not dropped by H_2_O_2_ at 1 mM in 30 min (Figure 6B). The sublethal level of H_2_O_2_ (0.5 mM) lowered the total glutathione pool by about 35% in 10 min, but afterwards, it recovered (Figure 6A). In contrast, 0.2 mM acrolein, a lethal level, consumed 90% glutathione in 10 min, as observed previously [20]. The lethal level of H_2_O_2_, although, induced 68% cell death, but only 10% total GSH pool was decreased in 10 min, and in 60-min incubation, the total GSH pool was not decreased, rather showing an increasing trend. The result suggests that glutathione pool size and GSH reduction ratio are not directly correlated to the initiation of PCD in H_2_O_2_-stimulated cells. 

The changes of Asc content and Asc reduction ratio (Asc/Asc + dehydroascorbate (DHA)) was also measured after treatment with H_2_O_2_ and acrolein. The lethal level of H_2_O_2_ (1 mM) decreased an insignificant level of Asc ca. 12% in 10 min, and the sublethal level of H_2_O_2_ (0.5 mM) did not show any adverse effects on Asc level (Figure 6C). The Asc reduction ratio was also slightly decreased and recovered afterwards (Figure 6D). In contrast, the lethal level of acrolein, i.e., 0.2 mM consumed 70% Asc content in 10 min, as observed previously [20]. These results suggest that the lethal dose of H_2_O_2_ induced two-third percentage cells death without affecting cellular redox status. Thus, the mechanism of carbonyls accumulation is critical for cell viability. 

### 3.7. H_2_O_2_ Stimulus Regulates a Carbonyl-Scavenging Capacity in Tobacco BY-2 Cells 

**Figure 7:**
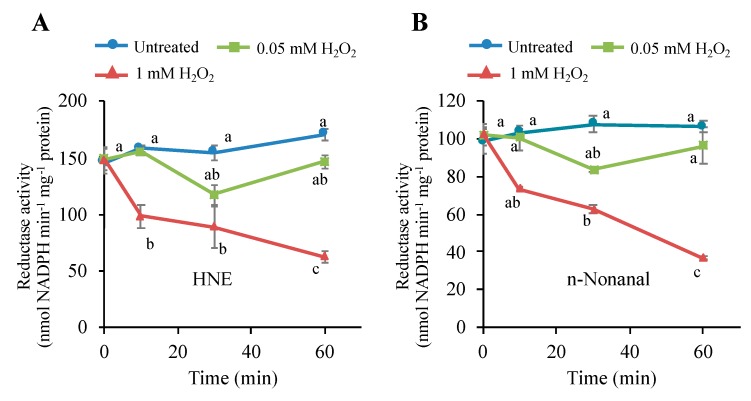
Effects of H_2_O_2_ on the NADPH-dependent HNE-reducing and *n*-nonanal-reducing activities in tobacco BY-2 cells. Four-d cultured cells were treated with H_2_O_2_ at 0.5 mM and 1 mM, as in Figure 1. Then, cells were harvested at the indicated time point, and proteins were extracted as in the Materials and Methods section. The reductase activities for (**A**) HNE and (**B**) *n*-nonanal were determined as in the Materials and Methods section. Each point represents the mean of three independent experiments and the error bars of the SEM. Different letters represent significantly different values (*p* < 0.05 on Tukey test).

In BY-2 cells stimulated with H_2_O_2_, only limited types of carbonyls were increased early (Figure 5). This was rather unexpected from our previous observation that, in oxidative stressed plant cells, many types of oxylipin carbonyl, including various RCS, are produced [12,31]. This contradiction lead us to conceive the presence of a regulation mechanism of carbonyl increase. We conceived a possibility that H_2_O_2_ attenuated certain types of carbonyl-scavenging enzyme and eventually increased the levels of limited types of carbonyls, especially HNE. Plant cells have NAD(P)H-dependent HNE-scavenging reductases, including 2-alkenal reductase (AER) [37,38] and aldo-keto reductase [13,14,39]. We treated BY-2 cells with lethal and sublethal doses of H_2_O_2_, and then extracted proteins and determined the NADPH-dependent HNE reductase activity (Figure 7A). It was found that the H_2_O_2_ treatment at a lethal dose (1 mM) decreased the reductase activity by 35% in 10 min and by 60% in 60 min. Sublethal H_2_O_2_ (0.5 mM) treatment caused a slight but insignificant decrease in the reductase activity. Thus, both the accumulation of HNE and the suppression of the HNE-reducing enzyme activity were observed only in the cells undergoing PCD. This result suggested that the inactivation of HNE-reducing enzyme by the lethal level of H_2_O_2_ was a cause of the enhancement of the intracellular HNE level, which eventually leads to PCD. 

To characterize the inactivated reductase, we also measured the NADPH-dependent activity for the saturated aldehyde *n*-nonanal. The *n*-nonanal-reducing activity responded to H_2_O_2_ treatment similar to the HNE-reducing activity; it was quickly decreased in the lethal level of H_2_O_2_, and in the sublethal level, it was decreased slightly and recovered afterward (Figure 7B). These results suggest that the H_2_O_2_-sensitive reductase had substrate specificity for both HNE and *n*-nonanal, i.e., it was a carbonyl reductase. Alternatively, there might be two different reductases for HNE and *n*-nonanal, and both showed similar sensitivity to H_2_O_2_.

### 3.8. H_2_O_2_ Directly Inactivates the Carbonyl Reductase 

**Figure 8:**
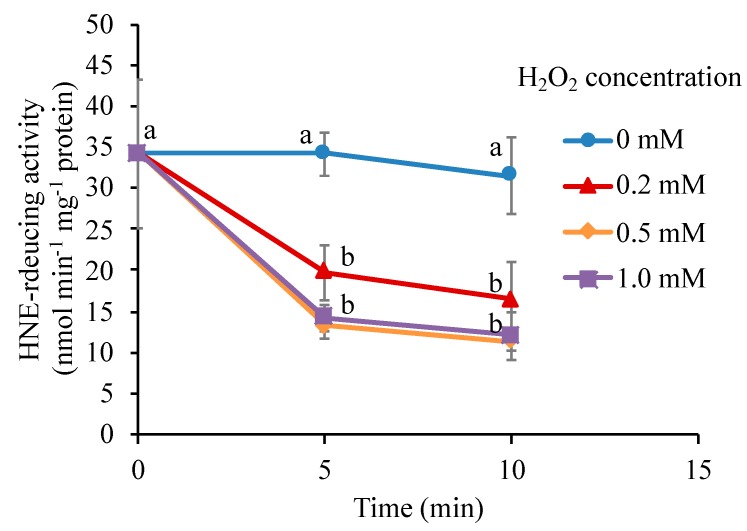
Effect of H_2_O_2_ on the NADPH-dependent HNE-reducing activity in protein extract from BY-2 cells. Proteins were extracted from untreated cells. H_2_O_2_ was added at the indicated concentration to the protein extract in the presence of 0.5 mM NH_2_OH. The HNE-reducing activity was determined at the indicated time after addition of H_2_O_2_, as in the Materials and Methods section. Different letters among the treatments represent significantly different values (*p* < 0.05 on Tukey test).

The addition of H_2_O_2_ to living cells will cause a broad range of biochemical changes, and the inactivation of the carbonyl reductase described above (Figure 7) could be a consequence of sequential reactions involving multiple proteins as observed in signal transduction. To verify the role of H_2_O_2_ in the inactivation of the carbonyl reductase, we prepared protein extract from untreated BY-2 cells and examined the effects of H_2_O_2_ addition on the NADPH-dependent HNE-reducing activity in it (Figure 8). In this experiment, we added 0.5 mM hydroxylamine to eliminate the catalase activity so that the added H_2_O_2_ was not immediately scavenged. It was found that H_2_O_2_ caused a rapid decrease in the HNE-reducing activity. In 5 min at 0.2 mM H_2_O_2_, the carbonyl reductase activity was lost by about half. Because the inactivation of HNE-reducing activity was observed in the cell-free protein extract, where intricate interactions among cellular components are very unlikely, we concluded that H_2_O_2_ directly acted on the carbonyl reductase and inactivated it.

**Figure 9:**
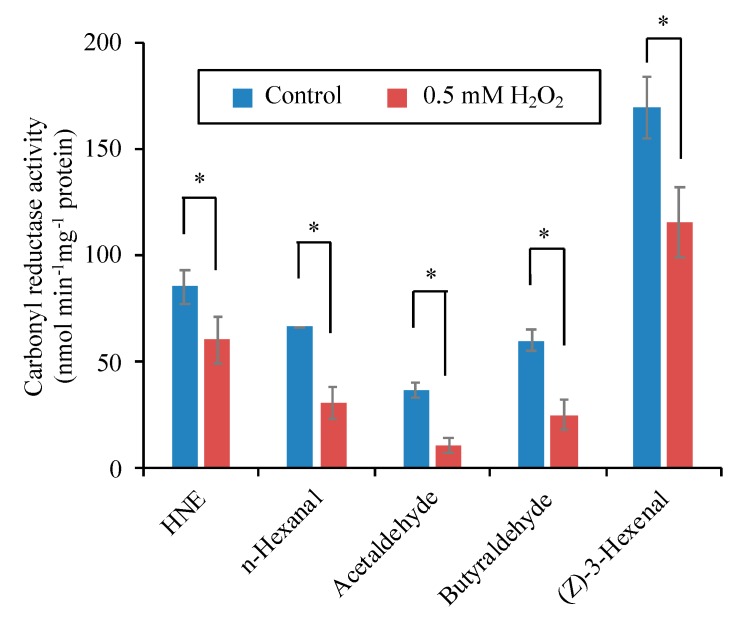
Effects of H_2_O_2_ on the NADPH-dependent carbonyl reductase activity for different substrates. Proteins were extracted from the untreated tobacco BY-2 cells. H_2_O_2_ at 0.5 mM was added to the cell extracts, and after 10-min incubation, the enzyme activity for the indicated substrate were determined as described in the Materials and Methods section. Each point represents the mean of three independent experiments and the error bars of the SEM. Asterisks indicate a statistical difference (*p* < 0.05, Student’s *t*-test).

Because H_2_O_2_ treatment of the cells caused inactivation of both HNE-reducing and *n*-nonanal-reducing activity, as shown in Figure 7, the H_2_O_2_-sensitive carbonyl reductase may show broad substrate specificity for various aldehydes. In the protein extract, we determined carbonyl reductase activity for five different aldehydes, i.e., HNE, *n*-hexanal, acetaldehyde, butyraldehyde, and (*Z*)-3-hexenal, and examined the effect of H_2_O_2_ treatment on distinct activities (Figure 9). After H_2_O_2_ treatment, the carbonyl reductase activity for all these substrates became significantly lower than those in the untreated control. These results suggest that the H_2_O_2_-sensitive NADPH-dependent carbonyl reductase possesses a broad substrate specificity.

## 4. Discussion

### 4.1. H_2_O_2_-Induced Inactivation of Carbonyl-Detoxifying Enzyme(s) Enhances the HNE Level and Triggers PCD

We recently reported that, in response to oxidative stimulus, plant cells generated RCS and that the generated RCS triggered PCD via activation of caspase-like proteases [20]. In this study, we investigated the biochemical mechanism of RCS accumulation at the early stage of oxidative stimulus. We found a larger fraction (70%) of tobacco BY-2 cells were dying in 5 h after the treatment with 1 mM H_2_O_2_. In exploring the early events caused by the lethal dose of H_2_O_2_, we found a 2.5-fold increase of C3LP activity even in 10-min incubation, while the increase in the C1LP activity was insignificant. Suppression of C3LP activity by the specific C3LP inhibitor protected cell death, but the C1LP inhibitor did not. We found a 10-min exposure of the cells to a lethal level of H_2_O_2_ increased only limited species of carbonyls, including highly reactive HNE and HHE. Importantly, the addition of H_2_O_2_ at lethal level decreased a carbonyl reductase activity in 10-min incubation. These findings suggest that the inactivation of the carbonyl reductase by H_2_O_2_ is the very initial step of the ROS-mediated PCD in BY-2 cells. 

As compared with the condition in living cells, where the HNE reductase was not inactivated by 0.5 mM H_2_O_2_ (Figure 7), in a cell-free solution, it was rather labile; H_2_O_2_ at 0.2 mM was high enough to affect the reductase activity (Figure 8). This difference is accounted for by the fact that living cells are equipped with the potent H_2_O_2_-scavenging system, including catalase and peroxidases. The addition of 0.5 mM H_2_O_2_ was not harmful to BY-2 cells because catalase and peroxidases with ascorbate scavenged H_2_O_2_ and thereby protected the carbonyl reductase. An excessive amount of H_2_O_2_ (1 mM<) could escape from the scavenging system, and the intracellular level of H_2_O_2_ would go over the threshold, to inactivate the carbonyl reductase. The inactivation profile of the carbonyl reductase in Figure 8, obtained in the presence of a strong inhibitor of heme-type enzymes, likely represented the intrinsic H_2_O_2_ sensitivity of the enzyme. Attenuation of the carbonyl reductase indirectly activates C3LP for the initiation of PCD. Therefore, the carbonyl reductase acts as a sensor of H_2_O_2_.

### 4.2. Consumption of Antioxidants Is Not a Major Cause of PCD

In our previous work, when PCD in BY-2 cells was started by the addition of acrolein, we observed a rapid drop of glutathione pool and significant decrease in the ascorbate pool, along with the activation of caspase-like proteases [20]. This posed a possibility that the loss of these antioxidants by RCS could also be a critical factor in causing PCD. In this work, we analyzed changes in the cellular redox status in response to H_2_O_2_ at different levels. We found that the effects of H_2_O_2_ of the lethal and sublethal doses were not different on the pool size and reduction level of both glutathione and ascorbate (Figure 6). Addition of H_2_O_2_ lowered the glutathione pool size slightly, but it was recovered in 60 min. Importantly, even though glutathione level was restored in 1 mM H_2_O_2_, PCD was not stopped (Figure 1). These results demonstrate that the main cause of PCD in H_2_O_2_-stimulated cells was not the redox status changes and supports the central role of RCS as an executor of PCD.

### 4.3. Candidates of the H_2_O_2_-Sensitive Carbonyl Reductase 

The protein of the H_2_O_2_-sensing carbonyl reductase in BY-2 cells has not been identified, but several characters of it have been revealed in this study: (i) Its activity depends on NADPH. (ii) It recognizes both HNE and *n*-nonanal as a substrate, i.e., it reduces the carbonyl moiety. The enzyme that has these properties is aldo/keto reductase (AKR). In plants and animals, and in the microorganism, 16 families and above 190 members of reductases present in the AKR superfamily to catabolize RCS. The identified AKR in higher plants classified in four families depending on their functions; detoxify reactive carbonyls, osmoprotectants, secondary metabolism, and membrane transport. In addition to the four families, AKR have three subfamilies; 4A, 4B, and 4C [14,40,41]. There are only limited number of isozymes thus far characterized of the substrate specificity [40,41] and, to our knowledge, there are no reports of their sensitivity to H_2_O_2_. 

In cell-free extract broad range of substrates from RCS, such as HNE, to less reactive carbonyls, such as *n*-hexanal, acetaldehyde, and butyraldehyde, (*Z*)-3-Hexenal showed H_2_O_2_ at 0.5 mM significantly reduced aldehyde reducing activity (Figure 7). If all these activities are ascribed to a single species of protein, the carbonyl reductase has a broad substrate specificity. Alternatively, it is possible that several different isozymes of carbonyl reductase participated, and they all were sensitive to H_2_O_2_. Purification of the carbonyl reductase will clarify this question.

### 4.4. Mechanism of the Inactivation of the Carbonyl Reductase by H_2_O_2_

ROS can oxidize various amino acid residues on a protein. Cys residue is especially sensitive to the oxidation and indeed such oxidative modification generally changes the normal activity or function of the protein [42]. For example, in response to H_2_O_2_, Cys199 moiety of OxyR, a six cysteine residues protein, oxidized to sulfenic acid and destabilized its activity. Cys199 modification accelerates to form a disulfide bond with Cys208, which enhances conformational changes of global OxyR and becomes susceptible to oxidation. A site-directed mutant C199S OxyR lost oxidative response and did not activate in the presence of H_2_O_2_ [43]. Peroxiredoxin (Prx) are ubiquitous enzymes appear in all types of organisms and metabolize peroxide to maintain cellular redox balance. The enzymatic activity of Prxs largely depends on the cysteine residues. The Prx family consists of three cysteine subfamilies, viz. 2-Cys, atypical 2-Cys, and 1-Cys. In human cell, 90% of the H_2_O_2_ generated in mitochondria reacts with Prx [44]. Thus, it is expected that the thiol moiety of the carbonyl reductase to be sensitive to oxidative signal. H_2_O_2_ sensors in the AKR family in plants have not been characterized yet. To elucidate the inactivation mechanism, we are trying to purify the H_2_O_2_-sensing carbonyl reductase.

## 5. Conclusions

In response to oxidative stimulus by H_2_O_2_, plant cells significantly increased C3LP activity and accumulate limited species of carbonyls, including RCS. The addition of H_2_O_2_ did not notably disturb redox balance of the cells but inactivated NADPH-dependent carbonyl reductase(s) in the cells and cell-free extract. Eventually, the carbonyls load increased and cells underwent PCD. Our results show a new mechanism of ROS sensing in plants: ROS is sensed by the carbonyl reductase(s), and its oxidative inactivation eventually increases carbonyls load and initiates the program of cell death. 

## Supplementary Materials

The following are available online at https://www.mdpi.com/2076-3921/9/2/141/s1: Figure S1. Acrolein-induced cell death was suppressed by the C3LP inhibitor. Four-d cultured BY-2 cells were treated with 0.2 mM acrolein or 0.2 mM acrolein + 20 µM of each inhibitor (C1LP or C3LP) and harvested after 5 h. (A) Typical fluorescence photographs (top row) and phase contrast microscopy of the same images (bottom): (i) untreated cells as control, (ii) 0.2 mM acrolein, (iii) 0.2 mM acrolein + 20 µM C3LP inhibitor, (iv) 0.2 mM acrolein + 20 µM C1LP inhibitor (top). White arrows indicate dead cells. (B) FDA-staining cells were counted for living cells and expressed as percentage. The data are the mean ± SE. Different letters represent significant different data (*p* < 0.05 on Tukey test). Figure S2: Typical chromatograms of DNP-carbonyls in BY-2 cells. The cells were treated with water as control (A) and H_2_O_2_ (B). Four-d-cultured cells treated with H_2_O_2_ at 1 mM and incubation for 10 min. The identified aldehydes are labelled at the top of each peak. Figure S3. Activation of C3LP in BY-2 cells after exposure to acetaldehyde. Four-d cultured cells were treated with various concentration of acetaldehyde as indicated. Protease inhibitors were added to cell extracts to a final concentration of 0.1 mM and caspase assay was performed as described in Materials and Methods. Statistical differences compared to controls are indicated by asterisks. (*p* < 0.05, Student’s *t*-test).
