# Correction: Biswas, M.S. et al. Inactivation of Carbonyl-Detoxifying Enzymes by H_2_O_2_ Is a Trigger to Increase Carbonyl Load for Initiating Programmed Cell Death in Plants. *Antioxidants* 2020, *9*, 141

**DOI:** 10.3390/antiox9040289

**Published:** 2020-03-31

**Authors:** Md. Sanaullah Biswas, Ryota Terada, Jun’ichi Mano

**Affiliations:** 1 Department of Horticulture, Bangabandhu Sheikh Mujibur Rahman Agricultural University, Gazipur 1706, Bangladesh; 2 Faculty of Agriculture, Yamaguchi University, Yoshida 1677-1, Yamaguchi 753-8515, Japan; y.rt.univ028.ge78@gmail.com; 3 Science Research Center, Organization of Research Initiatives, Yamaguchi University, Yamaguchi 753-8511, Japan; 4 Graduate School of Science and Technology for Innovation, Yamaguchi University, Yamaguchi 753-8511, Japan

The author wishes to make the following correction to this paper [1]. The H_2_O_2_ concentration of one experimental condition was mistyped in the index in Figure 7A,B. The correct Figure 7 is as follows:

These changes have no material impact on the discussion and conclusions of the paper. The authors would like to apologize for any inconvenience caused to the readers by these changes.

## Figures and Tables

**Figure 7:**
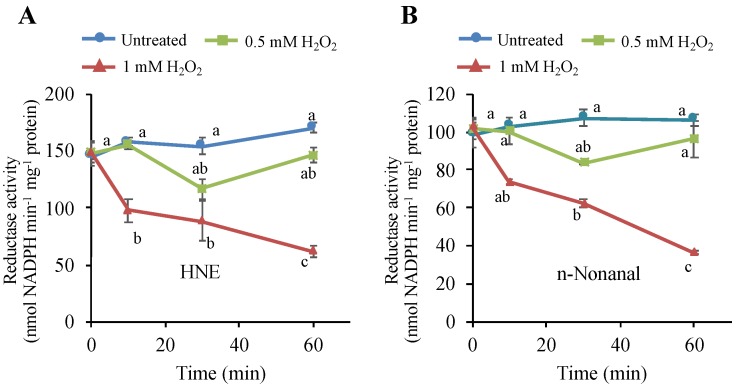
Effects of H_2_O_2_ on the NADPH-dependent HNE-reducing and *n*-nonanal-reducing activities in tobacco BY-2 cells. Four-d cultured cells were treated with H_2_O_2_ at 0.5 mM and 1 mM, as in Figure 1. Then, cells were harvested at the indicated time point, and proteins were extracted as in the Materials and Methods section. The reductase activities for (**A**) HNE and (**B**) *n*-nonanal were determined as in the Materials and Methods section. Each point represents the mean of three independent experiments and the error bars of the SEM. Different letters represent significantly different values (*p* < 0.05 on Tukey test).

## References

[1] Biswas M.S., Terada R., Mano J. (2020). Inactivation of Carbonyl-Detoxifying Enzymes by H_2_O_2_ Is a Trigger to Increase Carbonyl Load for Initiating Programmed Cell Death in Plants. Antioxidants.

