# Supplementary material for: Inactivation of Carbonyl-Detoxifying Enzymes by H2O2 Is a Trigger to Increase Carbonyl Load for Initiating Programmed Cell Death in Plants
Source: Antioxidants (Basel). 2020 Feb 6;9(2):141. doi: 10.3390/antiox9020141 (PMC7070697; doi:10.3390/antiox9020141)
Supplement: Supplementary file 1 [file antioxidants-09-00141-s001.zip › Figure_S1.pdf]

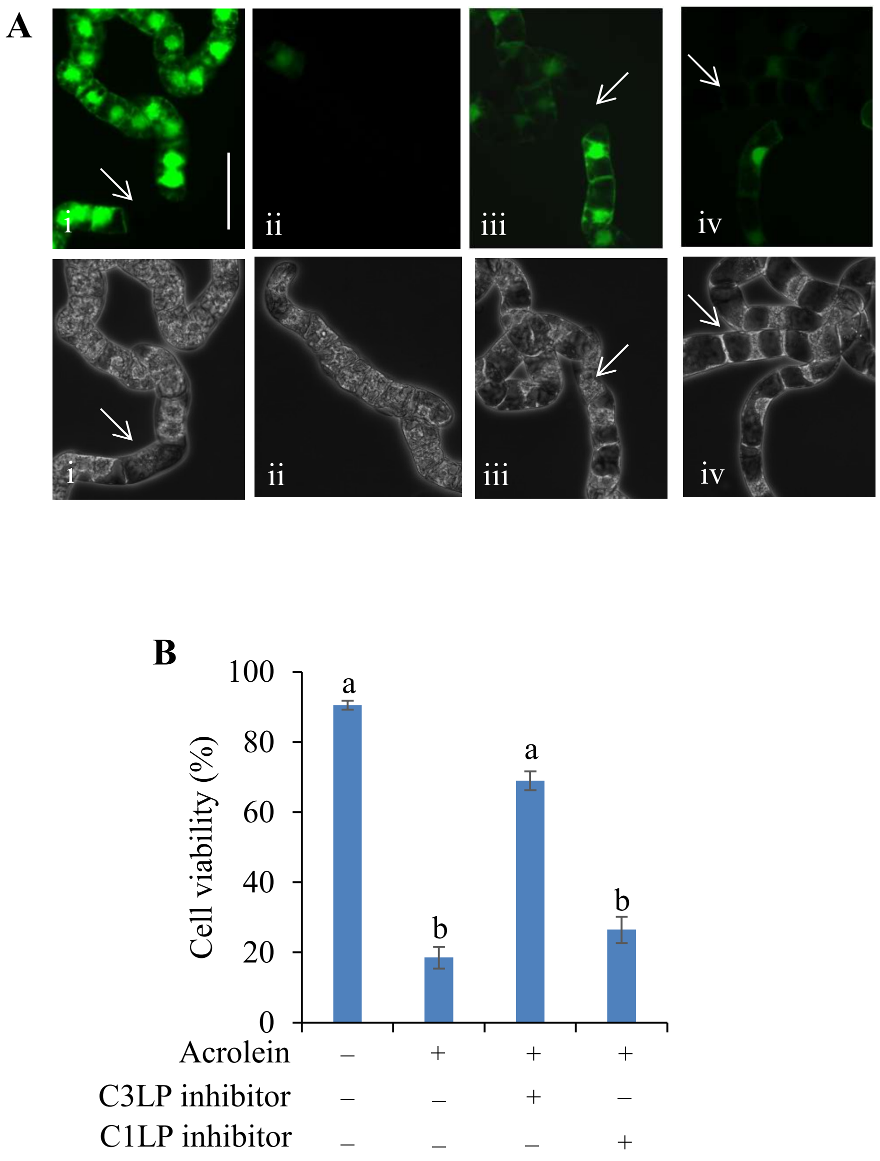

Figure S1. Acrolein-induced cell death was suppressed by the C3LP inhibitor. Four-d cultured BY-2 cells were treated with 0.2 mM acrolein or 0.2 mM acrolein + 20 µM of each inhibitor (C1LP or C3LP) and harvested after 5 h. (A) Typical fluorescence photographs (top row) and phase contrast microscopy of the same images (bottom): (i) untreated cells as control, (ii) 0.2 mM acrolein, (iii) 0.2 mM acrolein + 20 µM C3LP inhibitor, (iv) 0.2 mM acrolein + 20 µM C1LP inhibitor (top). White arrows indicate dead cells. (B) FDA-staining cells were counted for living cells and expressed as percentage. Mean of 3 runs ± SEM. Differences among treatments were analyzed by Tukey test. $P<0.05$.
