# Supplementary material for: Inactivation of Carbonyl-Detoxifying Enzymes by H2O2 Is a Trigger to Increase Carbonyl Load for Initiating Programmed Cell Death in Plants
Source: Antioxidants (Basel). 2020 Feb 6;9(2):141. doi: 10.3390/antiox9020141 (PMC7070697; doi:10.3390/antiox9020141)
Supplement: Supplementary file 1 [file antioxidants-09-00141-s001.zip › Figure_S2.pdf]

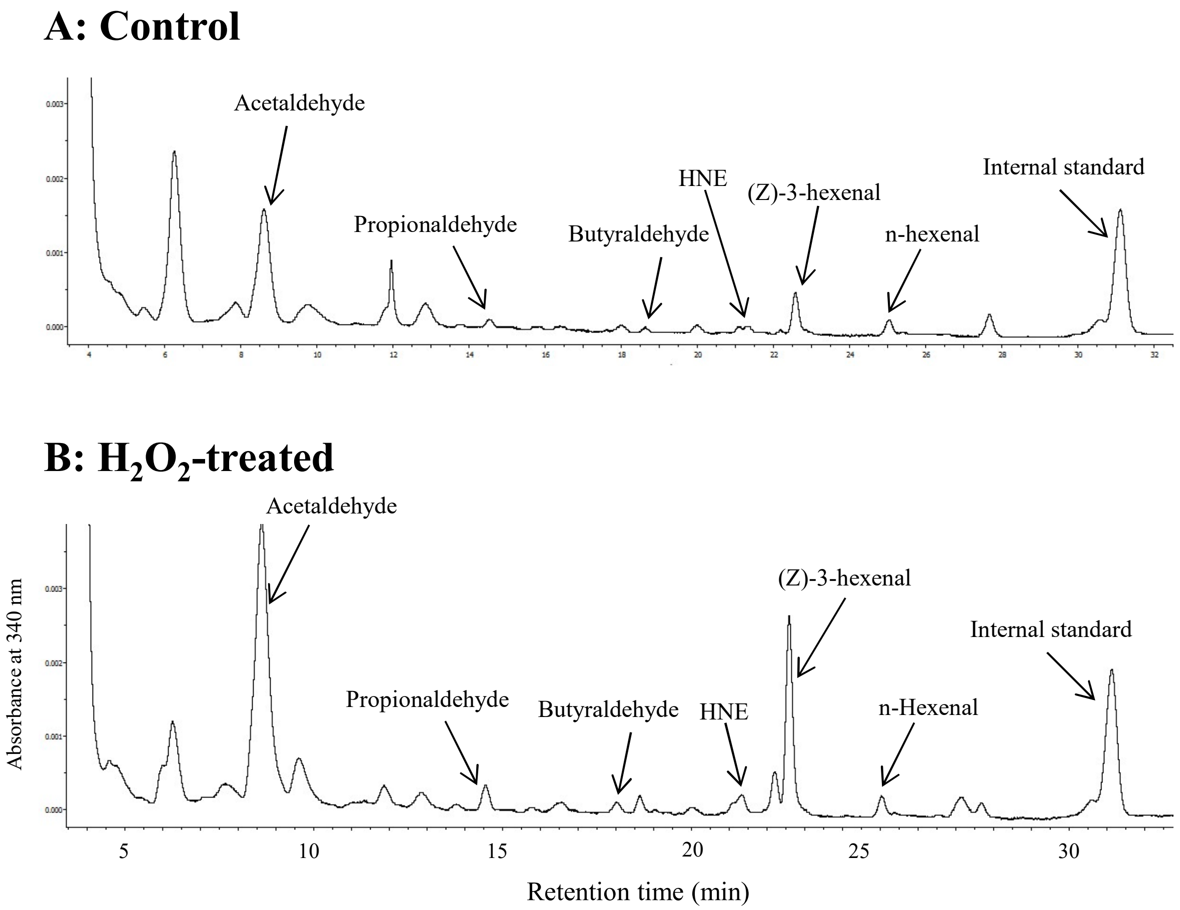

Figure S2. Typical chromatograms of DNP-carbonyls in BY-2 cells. The cells were treated with water as control (A) and H_2O_2 (B). Four-d-cultured cells treated with H_2O_2 at 1 mM and incubation for 10 min. The identified aldehydes are labelled at the top of each peak.
