# Supplementary material for: Inactivation of Carbonyl-Detoxifying Enzymes by H2O2 Is a Trigger to Increase Carbonyl Load for Initiating Programmed Cell Death in Plants
Source: Antioxidants (Basel). 2020 Feb 6;9(2):141. doi: 10.3390/antiox9020141 (PMC7070697; doi:10.3390/antiox9020141)
Supplement: Supplementary file 1 [file antioxidants-09-00141-s001.zip › Figure_S3.pdf]

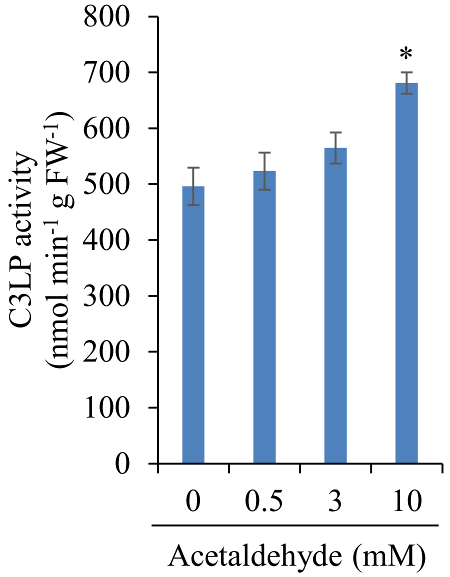

Figure S3. Activation of C3LP in BY-2 cells after exposure to acetaldehyde. Four-d cultured cells were treated with various concentration of acetaldehyde as indicated. Protease inhibitors were added to cell extracts to a final concentration of 0.1 mM and caspase assay was performed as described in Materials and Methods. Statistical differences compared to controls are indicated by asterisks. (*$P<0.05$, Student's *t*-test).
